# Circulating Melanoma Cell Numbers Correlate with TIGIT-Positive Cytotoxic T Cell Counts in Advanced-Stage Melanoma Patients

**DOI:** 10.3390/cells12060856

**Published:** 2023-03-09

**Authors:** Paula Kamińska, Karolina Buszka, Łukasz Galus, Maurycy Jankowski, Michał Nowicki, Jacek Mackiewicz, Mariusz Kaczmarek, Joanna Budna-Tukan

**Affiliations:** 1 Department of Histology and Embryology, Poznan University of Medical Sciences, 60-781 Poznan, Poland; 2 Doctoral School, Poznan University of Medical Sciences, 60-812 Poznan, Poland; 3 Department of Medical and Experimental Oncology, Heliodor Swiecicki University Hospital, Poznan University of Medical Sciences, 60-780 Poznan, Poland; 4 Department of Computer Science and Statistics, Poznan University of Medical Sciences, 60-806 Poznan, Poland; 5 Department of Cancer Immunology, Poznan University of Medical Sciences, 61-866 Poznan, Poland; 6 Department of Diagnostics and Cancer Immunology, Greater Poland Cancer Centre, 61-866 Poznan, Poland

**Keywords:** circulating melanoma cells, cytotoxic T cells, melanoma, immune checkpoints, diagnosis

## Abstract

Despite the rising public awareness of the risk factors and the possible prevention of melanoma development, it remains challenging in terms of diagnosis and treatment. To improve the clinical situation of patients, it would be especially beneficial to develop prognostic methods for the effective and continuous assessment of the disease course. The solution could lie in the selection of effective biomarkers derived from the tumor microenvironment, increasing the effectiveness of melanoma prognoses and monitoring. Hence, in this study, we evaluated the number of circulating melanoma cells (CMCs) in representative blood samples of melanoma patients vs. healthy controls, as well as the proportion of particular cytotoxic T cells in the total lymphocyte and leukocyte population as a reflection of immune resistance. The results were correlated with the clinical parameters of the patients to examine the potential value of CMC quantification and lymphoid cell phenotyping in melanoma diagnostics, prognostics, and treatment outcome monitoring. The CMC numbers were significantly higher in melanoma patients than in healthy controls. However, an analysis of the correlations between the baseline CMC counts and the clinical parameters found no significant results. In turn, we found significant differences between the groups in the percentage of various profiles of CD8+ cytotoxic T lymphocytes characterized by TIGIT and TIM-3 differential expression. Importantly, the CMC number correlated with CD8+TIGIT+ and CD8+TIGIT+TIM-3- cytotoxic T cell counts in the melanoma patient group. Considering the above, the combination of CMCs and the immunological status of the patient, as defined by the prevalence of selected immune cell types, seems to be a promising approach in melanoma diagnostics and prognostics.

## 1. Introduction

Despite the rising public awareness of the risk factors and the possible prevention of melanoma development, it remains challenging in terms of diagnosis and treatment. This malignancy arises as the result of abnormal melanocyte proliferation, and is characterized by a relatively high mortality rate. Individuals with a large number of melanocytic lesions, a pale phenotype, a positive family history of melanoma, and a high level of UV exposure are particularly prone to developing the disease [1]. To improve the clinical situation of patients, it is necessary to introduce rapid diagnosis methods and implement the most effective treatment method into routine patient management. It would be especially beneficial to develop prognostic methods for the assessment of the disease course, allowing for the monitoring of therapies, e.g., immunotherapy or targeted therapy, in a continuous and effective manner [2]. However, there are some important medical obstacles to achieving these goals, including the practical limitations of clinicopathologic features and the vague usefulness of prognostic and predictive biomarkers currently known and used in clinical diagnostics. Most of latter, e.g., lactate dehydrogenase (LDH), S100B, C-reactive protein, programmed cell death-1 (PD-1)/PD ligand-1 (PD-L1), and the immunoscore, are still characterized by inadequacies, including a sensitivity limited to late-stage disease [3].

The solution to this problem could lie in the selection of the most effective biomarker combinations, including those derived from the tumor microenvironment, thus increasing the effectiveness of melanoma prognoses and monitoring. The available data strongly indicate that circulating tumor cells (CTCs) can significantly enrich cancer diagnosis and treatment progress tracking. While the last decade has been dominated by data on CTCs in the context of prostate, lung, and colorectal cancer, some current publications have reported circulating melanoma cells (CMCs) as a tool for the effective and accurate profiling of this malignancy [4].

Like all CTCs, CMCs originate from the primary tumor and, after undergoing an epithelial–mesenchymal transition (EMT), migrate to tissues susceptible to metastasis formation via the circulatory system [5,6]. It is suggested that the count of CMCs in the bloodstream reflects the disease stage and susceptibility to metastasis formation. Circulating tumor cells are well understood in the medical and scientific community as the elements of the tumor microenvironment, but little is known about their exact effect on and/or crosstalk with other cells, including, but not limited to, the elements of the immune system.

Lymphocytes are one of the key players of the immune system, and the identification of their profile has already been shown to be useful in diagnostics and predicting the efficacy of immunotherapy in some cancers, including melanomas [7,8,9]. The well-known function of T lymphocytes is to distinguish normal cells from pathogens or tumor cells by activating or inactivating receptors on their surface. However, tumor cells are able to mimic the signals of normal cells, preventing inactivation by the immune system, and thus enabling further tumor growth [10]. In addition to CD4+ lymphocytes, CD8+ cells are also a key component of anti-tumor immunity. Following their differentiation into cytotoxic T cells (Tc) and migration to the tumor microenvironment, they prevent tumor growth via cytotoxicity exerted against neoplastic cells [11]. However, the persistent contact of lymphocytes with the developing tumor results in their exhaustion, which they manifest through the expression of various types of receptors called immune checkpoints (ICPs), leading to immune resistance.

The development of immunotherapy for various types of cancer has contributed to an increased interest in ICP identification. Immune checkpoints can stimulate or inhibit immune cell-related signals, regulating their function and maintaining homeostasis [12]. Among the profoundly studied cytotoxic T lymphocyte-associated antigen 4 (CTLA4) and programmed cell death protein 1 (PD1) [13], T cell immunoglobulin, the ITIM domain (TIGIT), and T-cell immunoglobulin mucin-3 (TIM-3) are worth mentioning. The role and interaction of these proteins have previously been described in the context of breast cancer [14].

**Figure 1:**
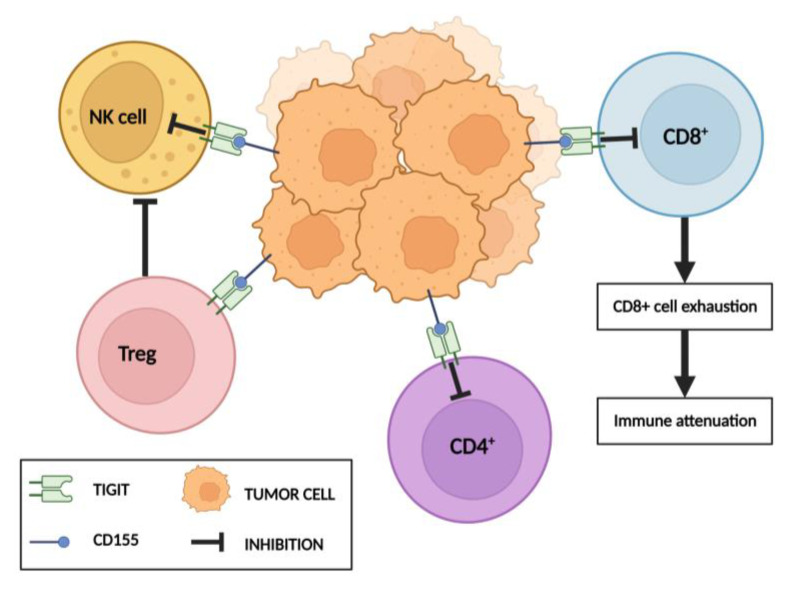
Mechanisms of TIGIT interaction with T lymphocytes and NK cells. TIGIT via its receptor CD155 triggers direct inhibitory signals in T lymphocytes and NK cells. Cytotoxic T lymphocytes undergo exhaustion. In turn, TIGIT signaling enhances immunosuppressive functions of Treg cells. Both actions consequently lead to immune system attenuation. Created with Biorender.com (accessed on 20 December 2022).

The TIGIT receptor protein, an immune checkpoint [15], is found in small quantities on the surface of naïve lymphoid cells. Moreover, natural killer cells (NK cells), Tc cells, T helper cells (Th cells), and T regulatory cells (Tregs) all cause its increased expression upon their activation [16,17]. A higher TIGIT expression has already been associated with the tumor microenvironment [18], correlating with metastasis and a poor prognosis [17]. As the result of various mechanisms (including the inhibition of T cell priming), TIGIT inhibits both T cells and NK cells, leading to an impaired anti-tumor immune response and making it an interesting factor in the context of cancer immunotherapy [17] (Figure 1). Moreover, the TIGIT blockade improves the function of cytotoxic lymphocytes [19]. The presence of this mechanism has been revealed in various tumor types, including non-small cell lung cancer (NSCLC), melanoma, advanced hepatocellular carcinoma (HCC), and pancreatic adenocarcinoma (PDAC) [18,20,21].

In turn, TIM-3 was first detected on the surface of Th cells, precisely Th1, mediating their apoptosis through binding to the galectin 9 ligand. TIM-3 is also present on the surface of macrophages, dendritic cells, and monocytes [22,23]. Importantly, its expression on CD8+ cells in the tumor microenvironment is thought to be a marker of cell dysfunction [24]. Similarly to TIGIT, TIM-3 promotes immune tolerance, and its overexpression is associated with a poor prognosis, making it a potential target for immunotherapy [25].

Tumor cells affect their external microenvironment, often modulating the function of the immune system through both the stimulation and inhibition of its various components. Therefore, an analysis of the changes in the phenotype of immune system cells after their contact with cancer cells could provide new insight into the mechanisms of malignancy development and spread. This knowledge, combined with CTC quantification, could contribute to the development of new diagnostic markers in various types of cancer. Hence, in this study, we evaluated the number of CMCs in representative blood samples of melanoma patients vs. healthy controls, as well as the proportion of particular cytotoxic T cells in the total lymphocyte and leukocyte population. The results were correlated with the clinical parameters of the patients to examine the potential value of CMC quantification and lymphoid cell phenotyping in melanoma diagnostics, prognostics, and treatment outcome monitoring.

## 2. Materials and Methods

### 2.1. Study Design

Blood was collected from qualified patients who gave their full, informed, written consent for participation in the study. A CMC count assessment and a cytometric evaluation of lymphoid cells were performed in parallel. One blood sample was collected before the start of the treatment (baseline).

### 2.2. Patient Characteristics

The study group consisted of 35 patients aged 37–88, in whom melanoma was confirmed through a histopathological evaluation of an excised skin lesion. The patients were recruited from the Department of Medical and Experimental Oncology, Heliodor Swiecicki University Hospital, Poznan University of Medical Sciences, Poznań, Poland, between 2019 and 2022. The sole inclusion criterion was the presence of melanoma, determined by a histopathological evaluation of the excised lesion. Patients were excluded from the study if they had other cancers; viral infections such as HIV, HCV etc.; or post-treatment complications.

After baseline blood sampling, the follow-up revealed CNS metastasis in 8 patients and liver metastasis in 7 patients. The *BRAF* mutation was detected in 18 patients. Progression occurred in 13 patients, of whom 5 died. The clinical data were consistent in terms of disease stage, LDH levels, lymphocytes, neutrophils, eosinophils, platelet counts, and the patient’s age (Table 1).

The control group consisted of 19 blood samples collected from age-matched, healthy individuals.

### 2.3. Circulating Melanoma Cell (CMC) Enumeration

**Figure 2:**
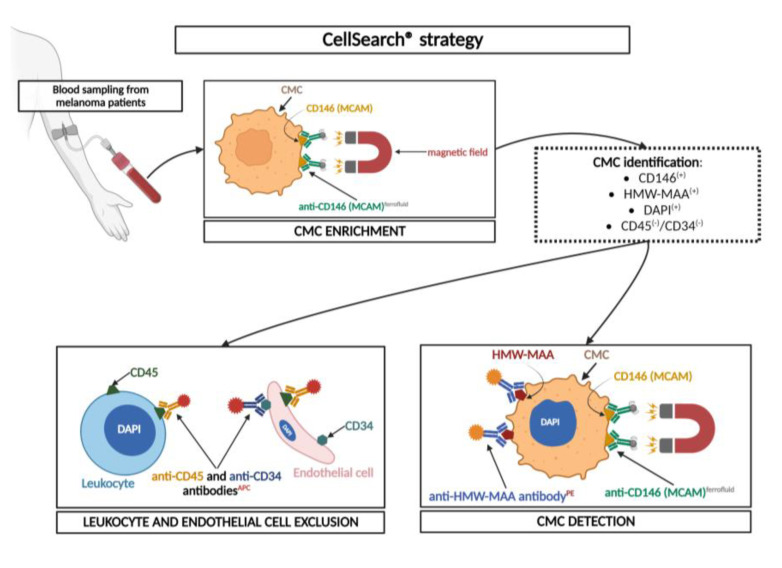
Principle of CellSearch^®^ strategy in patients with melanoma. The procedure was based on enrichment and detection steps. The positive CMC enrichment was based on the expression of CD146 by melanoma cells. Secondarily, CMC detection was based on the expression of the HMW-MAA and positive signals from the nucleus (stained with DAPI). Leukocytes and endothelial cells were excluded based on CD45 and CD34 marker expression (respectively). Abbreviations: MCAM, melanoma cell adhesion molecule; HMW-MAA, high molecular weight; PE, phycoerythrin; APC, allophycocyanin; DAPI, 4′,6-diamidino-2-phenylindole. Created with Biorender.com (accessed on 20 December 2022).

For CMC detection, 7.5 mL of blood was collected into CellSave^®^ preservative tubes (Silicon Biosystem, Menarini, Florence, Italy), ensuring cell viability for up to 96 h. Blood was analyzed within 24 h following the blood draw. To count the CMCs in the blood sample, the CellSearch^®^ system (CellTracks^®^ Autoprep^®^ System, CellTracks^®^ Analyzer II^®^ System, Silicon Biosystem, Menarini, Florence, Italy) and appropriate reagents (CellTracks Circulating Melanoma Cell Kit, Silicon Biosystem, Menarini, Florence, Italy) were used. The analysis was performed according to the manufacturer’s procedure. The method was based on the usage of magnetic beads (ferrofluid) coated with antibodies against the MCAM (CD146) antigen for CMC enrichment. In the next detection step, MCAM-positive cells were immunostained with antibodies against HMW-MAA (high molecular weight) PE, CD34-APC (endothelial marker), CD45-APC (leukocyte marker), and DAPI (nuclear staining). Only MCAM- and HMW-MAA-positive and CD34- and CD45-negative cells with an intact nuclear signal were identified as CMCs (Figure 2). The results are presented as the number of CMCs detected per 7.5 mL of blood [26,27]. An analysis of healthy individuals (control group) was performed to exclude false-positive signals.

According to the manufacturer’s recommendation, the CellTracks CEC/CMC Control Kit, containing the SK-Mel-28 cell line, was used (Silicon Biosystem, Menarini, Florence, Italy) as an internal control.

### 2.4. Flow Cytometry Assessment of Lymphoid Cells

To assess the number of lymphoid cells, precisely cytotoxic T cells with the expression of their specific antigens, the blood samples were analyzed using flow cytometry. For the phenotype determination of lymphocytes and the assessment of their proportions in the total population, the cells were stained with combinations of anti-CD14 PE/anti-CD45 FITC (IgG_2_α, IgG_1_, clone 2D1, MφP9), anti-CD3 APC-Cy7 (IgG_1_, clone SK7), anti-CD8 PE-Cy7 (IgG_1_, clone SK1), anti-TIGIT PerCP-Cy5.5 (IgG_2_b, clone 741182), and anti-TIM-3 APC (IgG_1_κ, clone 7D3) antibodies (Becton Dickinson, San Jose, CA, USA). The samples and unstained controls were further processed. Following incubation in darkness at room temperature, the erythrocytes in the samples were lysed and the bonds between antibodies and antigens were fixed. Next, the samples were washed twice in phosphate-buffered saline (PBS buffer) and subjected to data acquisition using a BD FACSAria^®^ Cell Sorter with a standard 6-color configuration (Becton Dickinson, USA). The analysis was performed on 5 × 10^4^ cells in the sample using the BD FACSDiva^®^ Software, version 6.1.2 (Becton Dickinson, USA). Leukocytes were defined based on the positive expression of the CD45 antigen and the negative expression of CD14. Among them, lymphocytes were identified based on their characteristic properties in the forward (FSC) and side (SSC) scatter and the positive expression of the CD3 antigen. For additional analyses, the gates were restricted to the CD3+CD8+ and CD3+CD8+TIGIT+TIM-3+ cells. The respective unstained controls were processed equally (Figure S1).

### 2.5. Statistical Analysis

The statistical analysis was performed using Dell Statistica (data analysis software system), version 13 (Dell Inc., 2016, Round Rock, TX, USA). Quantitative data were calculated as the mean, median, minimum, and maximum values, together with the standard deviation (SD). All results were first verified by a normality test (Shapiro–Wilk test). Since the test confirmed a lack of normality in some cases, the non-parametric Mann–Whitney U test was used to compare the results between groups. To compare the variables consistent with the normal distribution, an unpaired *t*-test was used. Moreover, the Spearman rank test was used to examine the correlations between the selected variables. A *p*-value of less than 0.05 was considered significant.

## 3. Results

### 3.1. Circulating Melanoma Cell Counts Obtained Using the CellSearch^®^ System

**Figure 3:**
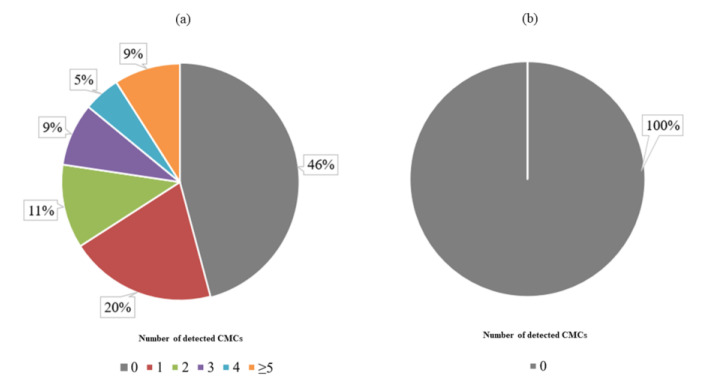
Distribution of detected circulating melanoma cells (CMCs) using the CellSearch^®^ system (**a**) among patients and (**b**) in the control group.

**Figure 4:**
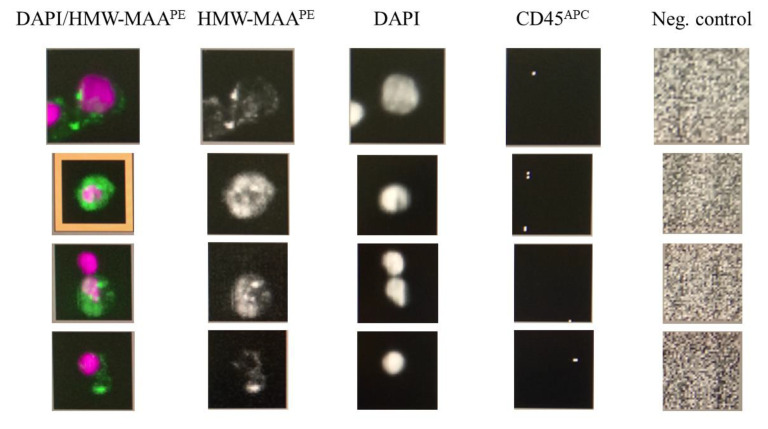
Representative images of CMCs detected in advanced-stage melanoma patients using the CellSearch^®^ system. Cells were identified as tumor cells according to the following criteria: HMW-MAA-positive, DAPI-positive, CD45-negative, and negative for the last channel.

The CellSearch*^®^* system analysis demonstrated that 54% (19/35) of patients were CMC-positive, with ≥1 CMC detected (range 0*–*8, median 1 (0;2)) (Figure 3a). In turn, we found no CMCs (0 CMC) in the control group of heathy individuals (Figure 3b). The difference between the melanoma patient and control group was significant (*p* = 0.041; non-parametric Mann–Whitney U test). Representative images of detected CMCs were presented in Figure 4.

### 3.2. Correlation between CMC Count and Clinical Parameters

An analysis of the correlations between the baseline CMC counts and some of the clinical parameters, precisely age, LDH level blood count, and cancer stage, found no significant results (Table 2; Spearman rank test). Similarly, we found no significant results between the CMC numbers compared to other clinical parameters, such as metastasis formation and the presence of the BRAF mutation (Table 2; Mann–Whitney U test).

### 3.3. Differences in Cytotoxic T Cell Profile in Melanoma Patients vs. Healthy Individuals

The results of the cytometric analysis of T lymphocytes, with a particular focus on cytotoxic T cells, revealed multiple statistically significant differences between melanoma patients and the control group of healthy individuals.

Primarily, the percentages of particular phenotypes in the population of all leukocytes were evaluated, with significant differences found in melanoma vs. control patients in CD3+ lymphocytes and CD3+CD8+ lymphocytes. All the percentage values were lower in melanoma patients than in healthy subjects (Table 3).

**Figure 5:**
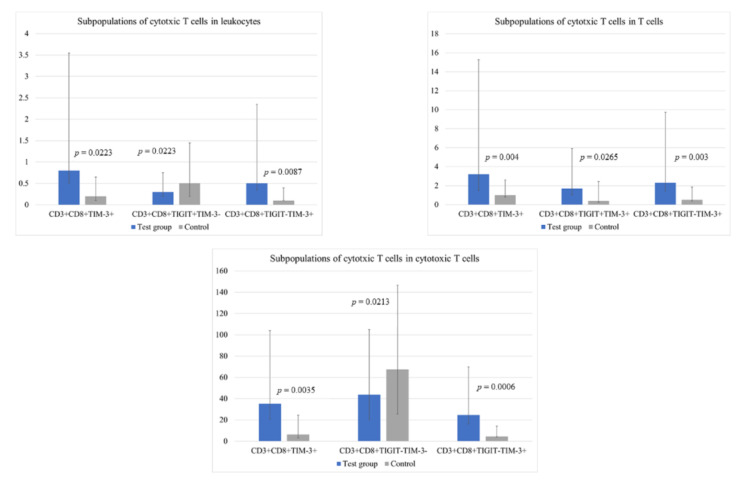
Bar graphs illustrating significant differences between groups in the percentage of certain subpopulations of cytotoxic T cells in the entire population of leukocytes, T cells, and cytotoxic T cells.

**Figure 6:**
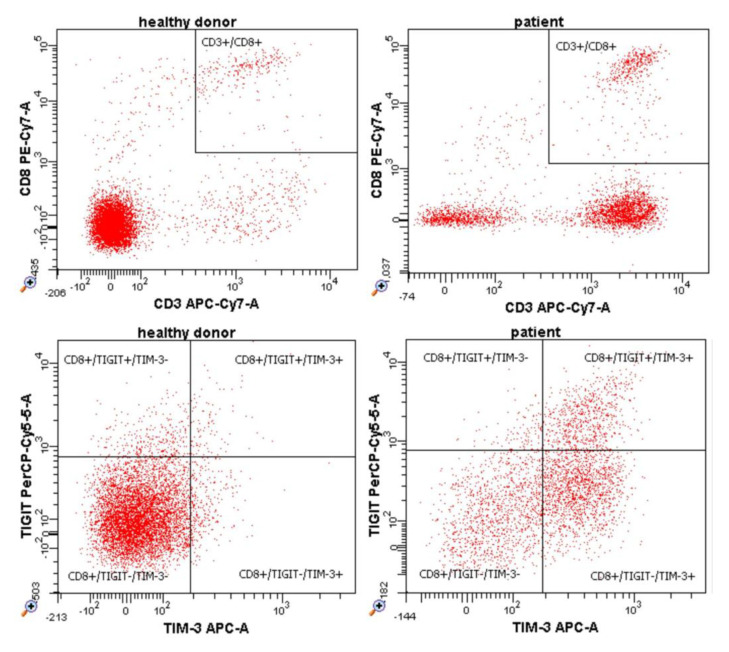
Representative example of flow cytometry dot plots presenting certain subpopulations of cytotoxic T cells in the entire population of cytotoxic T cells in healthy donors and melanoma patients.

Importantly, we found significant differences between groups in the percentage of various profiles of CD8+ cytotoxic T lymphocytes characterized by TIGIT and TIM-3 differential expression. Specific subpopulations and their percentage values are presented in Figure 5 and Table S1. A representative example of flow cytometry dot plots is shown in Figure 6.

### 3.4. CMC Correlation with the Number of Exhausted CD8+TIGIT+ Cytotoxic T Cells

**Figure 7:**
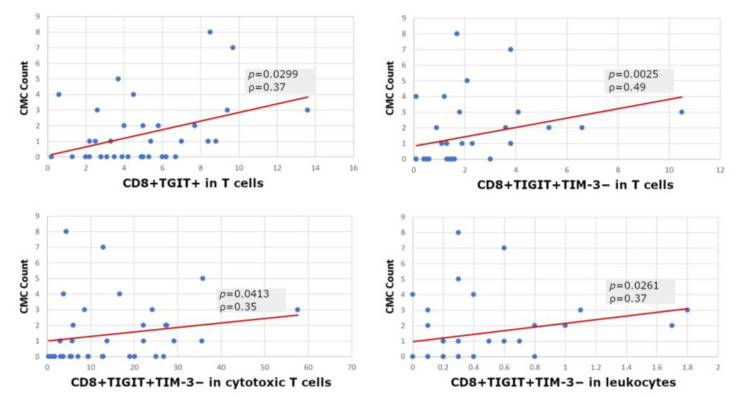
Correlations between baseline CMC counts and certain subpopulations of cytotoxic T cells (Spearman rank test).

We found a significant correlation between the number of detected CMCs and the prevalence of cytotoxic T cells expressing solely TIGIT (CD8+TIGIT+) within the population of T cells, and TIGIT with a lack of TIM-3 expression (CD8+TIGIT+TIM-3*−*) within the total population of leukocytes, T cells, and Tc cells in the patient group (Figure 7).

## 4. Discussion

Circulating tumor cells have been widely studied for over a decade, with the knowledge regarding their biology, transition, and clinical utility continuously expanding. Based on the results of breast [28], prostate [29,30], lung [31], and colorectal [32] cancer studies, it is known that CTC-based approaches often significantly improve the methods of cancer diagnosis and treatment monitoring. Research results are also promising in terms of CMC’s usefulness as a disease status biomarker for melanoma [33]. Moreover, according to some study outcomes, CMC numbers exhibit a significant prognostic value, enabling more effective monitoring of treatment effectiveness [34,35,36,37].

The CellSearch*^®^* system (Menarini), already approved by the Food And Drug Administration (FDA) for the diagnosis of breast, prostate, and colorectal cancers, also seems promising in the case of melanoma, with recovery rate of 88% [38]. This technology, customized to detect the melanoma cell adhesion molecule (MCAM), has already been shown to successfully predict the disease and monitor therapy outcomes [39]. Using CellSearch*^®^* technology, we found statistically significant differences in CMC counts between the study groups, with higher numbers in the blood of melanoma patients. Since no false-positive signals were detected in the control group of healthy individuals, it can be assumed that CellSearch*^®^* is an effective method for CMC detection. This stays in accordance with previous reports underlining the particular utility of CMC-based approaches in metastatic disease assessments, usually associated with a higher number of tumor-originating circulating cells [40,41], which could potentially translate into more effective therapy monitoring [40]. Mumford and Robertson compared different CMC isolation methods and found that the sensitivity of CellSearch*^®^*, ranging from 1 to 0.5 cells/mL, was superior to other methods such as density gradient centrifugation, immunomagnetic melanoma cell enrichment, ISET, and RARE [42]. Nevertheless, there are still several obstacles to effectively utilizing CMC, including the especially challenging aspects of the high heterogeneity of melanoma cells, even within one tumor [43], and the low concentration in the peripheral blood [33,44]. On the contrary, some authors have pointed out the inconveniences of the method, including the low sensitivity of the analysis and the need to use a wider antigen panel [45]. Hida et al. emphasized that determining CMC counts allows for the selection of a unique subgroup of metastatic patients. A combination of a CMC analysis with 5-S-cysteinyldopa (5-S-CD) resulted in a lower false-negative rate and a higher sensitivity than when using a single method. Thus, CMC quantification can potentially complement the effectiveness of standard metastasis detection approaches [45].

The results of other authors are mostly coherent with ours. We detected ≥1 CMC/7.5 mL blood in 54% of patients. This percentage is in line with the results of other researchers, in which the detection rate of CMC was around 40% [35]. Moreover, the lack of CMCs in the remaining group of stage IV melanoma patients may be explained by the pre-metastatic patient condition or a limited use of antigens in the CellSearch*^®^* method, which has been previously brought up by some authors [39]. The presence of CMCs was associated with progression in advanced melanoma patients. In the study by Freeman et al., differences in the number of CMCs in patients with and without metastases were found, suggesting their possible usefulness in determining disease progression [46]. In turn, Lucci et al. evaluated CMCs in 243 patients with stage III melanoma using the CellSearch*^®^* system. The detection of ≥1 CMCs was independently associated with disease recurrence, suggesting that CMC evaluation may be useful for identifying patients at risk of recurrence [47]. CMC quantification can also be used as a prognostic marker. Khoja et al. showed that patients with <2 CMCs exhibited a significantly longer median of overall survival (OS) than patients with ≥2 CMCs. Therefore, the authors suggested that a baseline CMC number of ≥2 could serve as an independent prognostic biomarker [35]. Similarly, in a study by Rao et al., patients with ≥2 cells detected were characterized by a significantly shorter OS compared to the group with less than two cells detected [38], which was further confirmed in other studies [45]. In turn, Hall et al. showed that the presence of one or more CMCs at the initial blood draw was correlated with a reduction in progression-free survival (PFS) in patients with stage IV melanoma [48]. In a study by Li et al., a large baseline CMC count was associated with deep local invasion, lymph node metastasis, and distant metastasis [41]. However, while the results of Roland et al. noted that CMCs were detected in 86% of patients with a stage IV disease compared to 29% of patients with a stage I disease, the CMC detection rate difference in stage II and III patients was not significant compared to stage IV patients [49], suggesting the need to include more patients in further studies.

In contrast to the results of our work, Panabieres et al. showed a correlation between LDH levels and CMC numbers, but similarly to us, did not note a correlation between the CMC number and the BRAF mutation [26]. The conclusions of Khoja et al. were similar, as the authors only detected a significant correlation of LDH with the number of CMCs among other clinical parameters [35].

CTC release and metastatic spread allows tumor cells to mimic normal cell signals, preventing immune system activation and, thus, enabling further tumor growth. This occurs, among other mechanisms, through the production of immunosuppressive factors and the recruitment of Tregs into the tumor microenvironment [10]. The presence of Tregs among tumor-infiltrating lymphocytes (TILs) is thought to be associated with a worse prognosis, while a Th and Tc cell presence is often correlated with better outcomes [50]. High levels of Tc cell-rich cell infiltration in melanoma patients are associated with a favorable prognosis [51]. However, cells in the tumor microenvironment are often characterized by abnormalities in cytokine production or cytotoxicity [52]. The impairment of the so-called functional exhaustion state of lymphocytes was associated with the expression of inhibitory receptors such as PD-1, CTLA-4, TIM-3 [53], and TIGIT [54], including on their surface. Hence, identifying the receptor expression profiles of cytotoxic lymphocytes could bring a significant benefit to the better determination of patient prognoses.

In our study, we found multiple differences between the study groups in the percentage of different Tc cell profiles, characterized by the presence or lack of TIGIT and TIM-3 expression, among the entire population of Tc cells. The CD8+TIM-3+, CD8+TIGIT+TIM-3+, CD8+TIGIT-TIM-3+, and CD8+TIGIT-*TIM-3−* subpopulations were found more frequently in melanoma patients compared to the control group, while CD8+TIGIT+*TIM-3−* presented an opposite pattern.

Interestingly, we found a statistically significant correlation between the number of CMCs detected and the prevalence of a CD8+TIGIT+ subpopulation in lymphocytes, and a CD8+TIGIT+*TIM-3−* subpopulation in Tc lymphocytes. These correlations prevailed when measured only in the cytotoxic T lymphocyte population, lymphocytes in general, or the entire sample in the patient group. Lee et al. showed that the high expression of TIGIT is associated with a worse survival and has a prognostic value for melanoma [16], which is consistent with our observations. In turn, in a study by He et al., patients with gastric cancer also showed an increased percentage of CD8+TIGIT+ T cells compared to healthy controls [55]. These cells exhibited functional exhaustion with impaired proliferation, activation, metabolism, and cytokine production. In the case of gastric cancer, as in pancreatic ductal adenocarcinoma (PDAC), the CD155/TIGIT axis was identified as a potential therapeutic target [21,55]. In a study by Liu et al., CD8+TIGIT+ T cells were associated with pathogenesis and the progression of patients with hepatitis B virus-related hepatocellular carcinoma [56]. In turn, in patients with muscle-invasive bladder cancer, that cell profile was also associated with a worse prognosis and immune failure [57]. Ostroumov et al. revealed that TIGIT has a key role in T-cell exhaustion in patients with liver cancer, and identified it as a potential target for checkpoint combination therapies [58]. Moreover, Iwahori et al. stated that in the case of immunotherapy, the increased cytotoxic activity of these T cells against cancer cells results in increased treatment effectiveness [11]. TIGIT expression identifies exhausted CD8+ T cells at different stages of their differentiation more reliably than PD-1. In the case of intrahepatic cholangiocarcinoma and hepatocellular carcinoma, patients can be divided into two subgroups on the basis of TIGIT expression in tumor-infiltrating CD8+ T cells, which is important for the choice of the right therapy [58]. Continuing this research, Chauvin et al. indicated that the simultaneous blockade of TIGIT and PD-1 should be considered as a means of achieving a strong antitumor response of CD8+ T cells in patients with advanced melanoma [54]. The dual blockade of PD-1/TIGIT increased the proliferation and function of tumor antigen-specific CD8+ T cells and TILs [19,54,59]. Due to the above, it is highly desirable to develop innovative technologies allowing for the specification of the total number, activity, and type of tumor antigen-specific cytotoxic T cells in the tumor microenvironment [11]. Flow cytometry, in combination with other methods, could be one of the methods with a significant application potential in such approaches.

A study by Blazkov et al. showed that CD8+TIGIT+ T cells are effective ex vivo in both HIV-aviremic patients and healthy donors. Hence, the authors doubted that all CD8+TIGIT+ T cells are subject to dysfunction and exhaustion [60]. Additionally, highly suppressive TIGIT+ Tregs were associated with a profound anti-tumor response, and some researchers hypothesize that the deprivation of TIGIT on Tregs, not on CD8+ TILs, enhances the anti-tumor response by reestablishing the cytotoxic properties of CD8+ T cells [61]. Both studies undermine the exceptional role of TIGIT+ Tc cells in uncontrolled tumor growth and spread, which may potentially explain our observation of a higher proportion of CD8+TIGIT-*TIM-3−* cells in melanoma patients vs. healthy individuals.

Unexpectedly, in our work, CMCs were not associated with the expression of TIM-3 on CD8+ T cells. Fourcade et al. demonstrated that TIM-3 (as well as PD-1) expression is associated with tumor antigen-specific CD8+ T cell dysfunction in melanoma patients. The authors indicated that it is reasonable to simultaneously employ a TIM-3-TIM-3L and PD-1-PD-L1 blockade to reverse tumor-induced T cell exhaustion/dysfunction in patients with advanced melanoma [62]. Moreover, Kurtulus et al. indicated that TIGIT and TIM-3 suppress the antitumor response synergistically, based on the observation that the exclusive TIGIT deficiency in CD8+ T cells does not contribute to the immune response, since compensation by other coinhibitory receptors, such as TIM-3, is likely [61]. Joller and Kuchroo supported this theory by pointing out that TIM-3 and TIGIT (as well as LAG-3) act in a cooperative manner, with PD-1 exerting essential inhibitory functions [63]. In our case, it can be speculated that, based on the cascade character of the activation axis, inhibitory TIGIT and TIM-3 molecules may also present sequential and complementary expression on T cells. During activation, different molecules appear successively on the surface of a lymphocyte, e.g.*,* first CD69, followed by CD25 and CD40L. Hence, when CD40L appears, CD69 may already be absent. Similarly, the exhausted cell state might also manifest through immune checkpoints. These may also be expressed at different times, depending on the severity of the exhaustion process, or preferentially by different subpopulations of lymphocytes. Moreover, according to Zhu et al. and Sanchez-Fueyo et al., TIM-3 only shows transient expression upon T cell activation, while stable expression is found only as a result of permanent stimulation [64,65].

Considering the above, there is certainly a need to provide an integrated and adequate set of therapeutic decision-facilitating biomarkers in melanoma. The combination of CMCs and the immunological status of the patient, defined by the prevalence of selected immune cell types, seems to be a promising approach. However, more studies are needed to fully grasp the extent to which this method can be applied in prognoses and therapy monitoring.

## Figures and Tables

**Table 1:** Clinical characteristics of melanoma patients participating in the study (*n* = 35).

Parameter		
		Median (Q1; Q3)
Age (years)		66 (59.5; 75.5)
LDH (U/L)		204 (189.75; 291.25)
Lymphocytes (10^9^/L)		1.77 (1.31; 2.23)
Neutrophils (10^9^/L)		4.54 (3.62; 6.7)
Eosinophils (10^9^/L)		0.15 (0.07; 0.19)
Platelets (10^9^/L)		263.5 (226.25; 301.75)
		*n* (%)
Cancer stage IV		35 (100)
Metastases	M1a	18 (51.4)
	M1b	4 (11.4)
	M1c	5 (14.3)
	M1d	8 (22.9)
*BRAF* mutation detected		18 (51.4)

LDH—lactate dehydrogenase; M1a—metastases in the skin, subcutaneous tissue, or lymph nodes; M1b—lung metastases without M1a; M1c—metastases in other organs without M1a and M1b, except metastases in the central nervous system; M1d—metastases in the central nervous system without M1a, M1b, and M1c.

**Table 2:** Dependencies between CMC counts and clinical parameters.

Parameter		Spearman’s Rank Correlation Coefficients			*p*
Age (years)		−0.05			0.7522
LDH (U/L)		0.17			0.3233
Lymphocytes (10^9^/L)		−0.03			0.8811
Neutrophils (10^9^/L)		0.13			0.4513
Eosinophils (10^9^/L)		−0.16			0.3784
Platelets (10^9^/L)		0.06			0.7538
Cancer stage		0.24			0.1708
		**Median (range)**	**Lower quartile**	**Upper quartile**	
Metastases in CNS	Absent	0 (0–7)	0	2	0.2061
	Present	2 (0–8)	0	4.5	
Metastases in liver	Absent	0 (0–7)	0	1.5	0.0872
	Present	3 (0–8)	0	5	
*BRAF* mutation	Absent	0 (0–8)	0	2	0.7078
	Present	1 (0–7)	0	2	

**Table 3:** Differences between study groups in the percentage of CD3+ and CD3+CD8+ lymphocytes in leukocyte population.

Population		Median (Q1; Q3)			Higher/Lower Values, Relative to the Control Group
		Test Group (*n* = 35) (%)	Control (*n* = 19) (%)	*p*	
**Leukocytes**	CD3+	19.63 (16.24; 24.08)	35.8 (31.51; 40.23)	0.0002	L
	CD3+CD8+	1.9 (1.15; 2.55)	3 (2.5; 3.8)	0.0137	L

## Data Availability

Not applicable.

## Supplementary Materials

The following supporting information can be downloaded at: https://www.mdpi.com/article/10.3390/cells12060856/s1, Figure S1: The gating strategy used during the evaluation of TIGIT and TIM-3 receptor expression on cytotoxic T cells (CD3+CD8+); Table S1: Differences between study groups in the percentage of various profiles of CD8+ cytotoxic T lymphocytes characterized by TIGIT and TIM-3 differential expression.
