# Supplementary material for: Circulating Melanoma Cell Numbers Correlate with TIGIT-Positive Cytotoxic T Cell Counts in Advanced-Stage Melanoma Patients
Source: Cells. 2023 Mar 9;12(6):856. doi: 10.3390/cells12060856 (PMC10047426; doi:10.3390/cells12060856)
Supplement: Supplementary file 1 [file cells-12-00856-s001.zip › cells-2208680-supplementary.pdf]

**Figure S1.** The gating strategy used during the evaluation of TIGIT and TIM-3 receptor expression on cytotoxic T cells (CD3+CD8+).

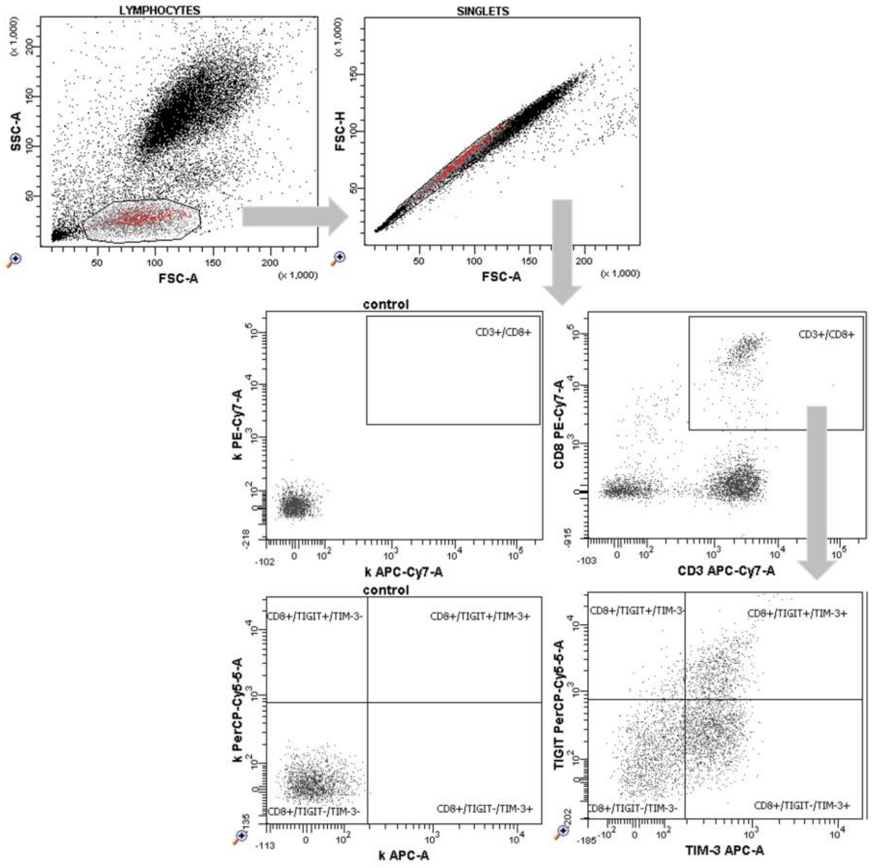


**Table S1.** Differences between study groups in the percentage of various profiles of CD8+ cytotoxic T lymphocytes characterized by TIGIT and TIM-3 differential expression.

| Population | | Median (Q1 ; Q3) | | | Higher/Lower values, relative to the control group |
|---|---|---|---|---|---|
| | | **Test group (N=35) [%]** | **Control (N=19) [%]** | ***p*** | |
| **Leukocytes** | CD8+TIM-3+ | 0.8 (0.3; 2.75) | 0.2 (0.1; 0.45) | 0.0223 | H |
| | CD8+TIGIT+TIM-3- | 0.3 (0.1; 0.45) | 0.5 (0.3; 0.95) | 0.0223 | L |
| | CD8+TIGIT-TIM-3+ | 0.5 (0.15; 1.85) | 0.1 (0; 0.3) | 0.0087 | H |
| **T cells** | CD3+CD8+TIM-3+ | 3.2 (1.7; 12.05) | 1 (0.2; 1.6) | 0.004 | H |
| | CD3+CD8+TIGIT+TIM-3+ | 1.7 (0.65; 4.2) | 0.4 (0.15; 2.05) | 0.0265 | H |
| | CD3+CD8+TIGIT-TIM-3+ | 2.3 (0.85; 7.45) | 0.5 (0.1; 1.35) | 0.0030 | H |
| **Cytoxic T cells** | CD8+TIM-3+ | 35.2 (14.2; 68.65) | 6.3 (3; 18.05) | 0.0035 | H |
| | CD8+TIGIT-TIM-3- | 43.7 (23.6; 61.3) | 67.5 (41.95; 79.3) | 0.0213 | L |
| | CD8+TIGIT-TIM-3+ | 24.6 (8.35; 45.2) | 4.5 (0.5; 9.8) | 0.0006 | H |
